# Does Melatonin Homeostasis Play a Role in Continuous Epigastric Pain Syndrome?

**DOI:** 10.3390/ijms140612550

**Published:** 2013-06-14

**Authors:** Cezary Chojnacki, Tomasz Poplawski, Janusz Blasiak, Jan Chojnacki, Grazyna Klupinska

**Affiliations:** 1 Department of Gastroenterology, Medical University of Lodz, 1 Haller’s Square, 90-647 Lodz, Poland; E-Mail: cezary.chojnacki@umed.lodz.pl; 2 Department of Molecular Genetics, Faculty of Biology and Environmental Protection, University of Lodz, Pomorska 141/143, 90-236 Lodz, Poland; E-Mails: tplas@biol.uni.lodz.pl (T.P.); jblasiak@biol.uni.lodz.pl (J.B.); 3 Department of Clinical Nutrition, Medical University of Lodz, 1 Haller’s Square, 90-647 Lodz, Poland; E-Mail: grazyna.klupinska@umed.lodz.pl

**Keywords:** continuous epigastric pain syndrome, AANAT, HIOMT, melatonin, 6-sulfatoxymelatonin

## Abstract

Two clinical forms of functional dyspepsia (FD) are listed in the Rome III criteria: postprandial distress syndrome (PDS) and epigastric pain syndrome (EPS), differing in the recurrence of ailments depending on the diet. Continuous EPS (CEPS) is observed in some EPS patients, also at night, but its cause is still unknown. We showed previously that melatonin (MEL) homeostasis may be associated with FD. In the present work we evaluated selected components of melatonin homeostasis in patients with CEPS. The study included 30 patients with CEPS, 21 women and nine men, aged 21–49 years and 30 control subjects (EPS excluded); organic and mental diseases, as well as *Helicobacter pylori* infection, were excluded in both groups. The average severity of abdominal pain in the last three months was estimated in a 10-point scale (Visual Analog Scale). The levels of mRNA expression of arylalkylamine-*N-*acetyltransferase (AANAT) and hydroxyindole-*O-*methyltransferase (HIOMT), the main components of MEL homeostasis, were determined in gastric mucosa with real time PCR. The fasting serum level of MEL (at 09:00 a.m.) and circadian urine excretion of 6-sulfatoxymelatonin (6-HMS) were determined with ELISA. AANAT expression in antral mucosa of control subjects was 1.76 ± 0.41, in the gastric body 1.35 ± 0.38, and in the dyspeptic group 1.42 ± 0.38 (*p* < 0.05) and 0.92 ± 0.55 (*p* < 0.05), respectively. HIOMT expression in the control was 2.05 ± 0.70 in the antrum and 1.57 ± 0.69 in the body and in the CEPS group, it was: 1.51 ± 0.57 (*p* < 0.05) and 0.74 ± 0.31 (*p* < 0.001), respectively. MEL concentration (pg/mL) was 9.41 ± 3.09 in the control group and 5.62 ± 1.34 (*p* < 0.01) in the CEPS group. Urinary 6-HMS excretion (μg/24 h) was 11.40 ± 4.46 in the controls and 7.68 ± 2.88 (*p* < 0.05) in the CEPS. Moreover, a negative correlation was found between the tested parameters and severity of epigastric pain. These results indicate that patients with CEPS may display low level of AANAT and HIOMT expression in gastric mucosa, resulting in decreased MEL synthesis.

## 1. Introduction

The gastrointestinal (GI) tract is a rich source of melatonin [1,2]. Exogenous l-tryptophan, found in many food products, is a substrate for the synthesis of this indoleamine. Tryptophan is converted to serotonin in enterochromaffin cells (ECs) with the involvement of arylalkylamine *N*-acetyltransferase (AANAT). Then, serotonin is converted to melatonin by 5-hydroxyindole-*O*-methyltransferase (HIOMT) [3]. Melatonin is absorbed from the GI tract and transported by the portal vein system to the liver where it is metabolized mainly to 6-hydroxymelatonin sulfate (6-HMS) [4,5]. Then 6-HMS is excreted with urine and its content in 24 h-urine collection is recognized as a good index of melatonin synthesis in pinealocytes and enterochromaffin cells [6,7]. The amount of melatonin derived from the GI tract depends mainly on the number of ECs and the expression of HIOMT, which catalyses the final step of its biosynthesis [8]. Melatonin affects function of many organs *via* membrane (MT1, MT2, MT3) and nuclear (RZR/ROR) receptors, but it also exerts a receptor-independent action. It binds to intracellular calmodulin affecting adenyl cyclase and phosphodiesterase [9,10].

Several experimental studies demonstrated enteroprotective property of melatonin [11–13]. It has been used in gastroenterology for therapeutic purposes in cases of its deficiency [14–16] or ineffectiveness of other treatments [17,18]. Functional dyspepsia (FD) belongs to diseases causing serious therapeutic problems. According to the Rome III criteria (2006) there are two distinguished clinical forms of this disease: postprandial distress syndrome (PDS) and epigastric pain syndrome (EPS) [19,20]. The main symptoms of PDS include early satiety, postprandial discomfort and nausea, in which prokinetic drugs provide relief [21]. In EPS epigastric pain occurs while fasting and between meals and proton-pump inhibitors (PPIs) are effective in this case [22].

Some patients do not meet the criteria because they experience persistent pain in the upper abdomen both before and after meals and frequently also at night. In these cases prokinetic drugs as well as PPIs are ineffective and alternative medications, other therapeutic procedures and new drugs are required. Melatonin is considered as a potential therapeutic agent in functional disorders of the GI tract [18,23].

The aim of our study was to evaluate the association between melatonin synthesis and its paracrine effect and CEPS in FD patients.

## 2. Results and Discussion

**Figure 1:**
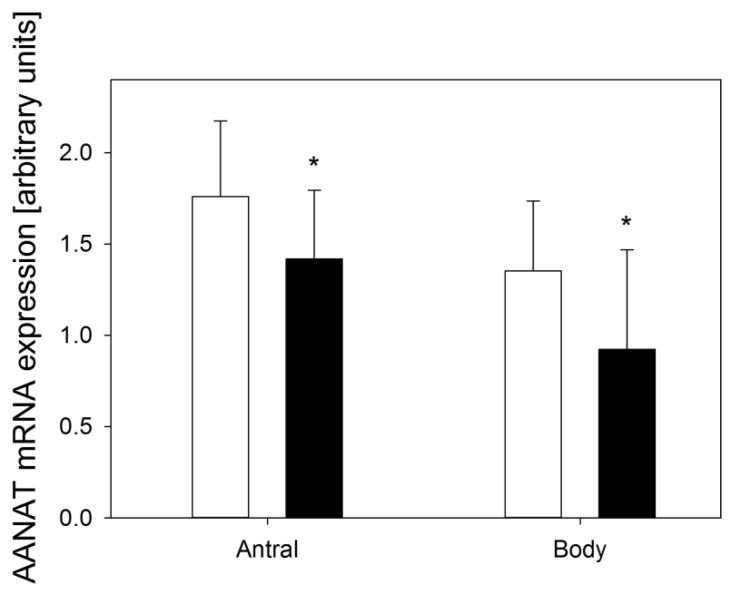
Expression of arylalkylamine-*N*-acetyltransferase (AANAT) assessed as relative quantity of mRNA of AANAT hypoxanthine phosphoribosyltransferase (reference) by RT-PCR in antral and body gastric mucosa in control subjects (white bars) and patients with continuous epigastric pain syndrome (black bars). *n* = 30 in either group; error bars denote standard deviation; *p < 0.05 compared with control.

In healthy subjects AANAT expression in antral mucosa was 1.76 ± 0.41, in the gastric body 1.35 ± 0.38 and in the dyspeptic group (CEPS) 1.48 ± 0.38 (*p* < 0.05) and 0.92 ± 0.55 (*p* < 0.05) (Figure 1), respectively.

**Figure 2:**
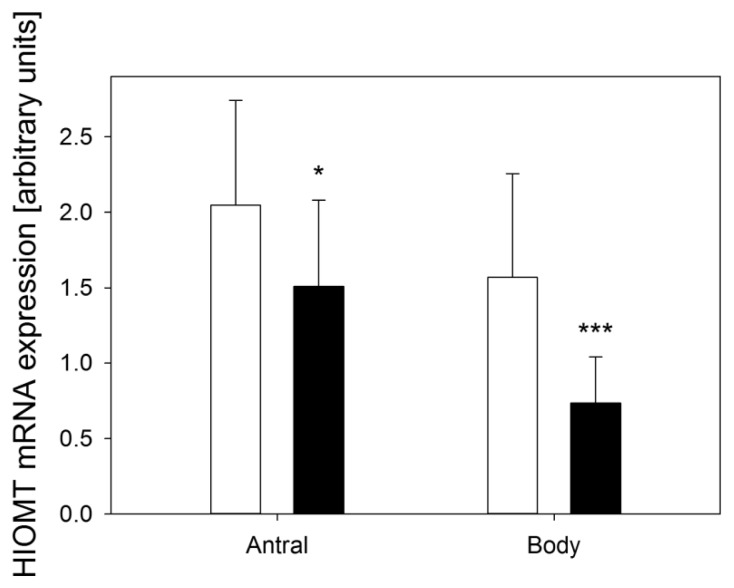
Expression of hydroxyindole-*O*-methyltransferase (HIOMT) assessed as relative mRNA quantity of HIOMT and hypoxanthine phosphoribosyltransferase (reference) by RT-PCR in antral and body gastric mucosa in control subjects (white bars) and patients with continuous epigastric pain syndrome (black bars). *n* = 30 in either group; error bars denote standard deviation; *p < 0.05, ***p < 0.001 compared with control.

In the control group HIOMT expression in antral mucosa was 2.05 ± 0.70 and in the gastric body 1.57 ± 0.69. Expression of this enzyme in patients with dyspepsia (CEPS) was lower; in the antrum it was 1.51 ± 0.57 (*p* < 0.05), and in the gastric body 0.74 ± 0.31 (*p* < 0.001) (Figure 2).

**Figure 3:**
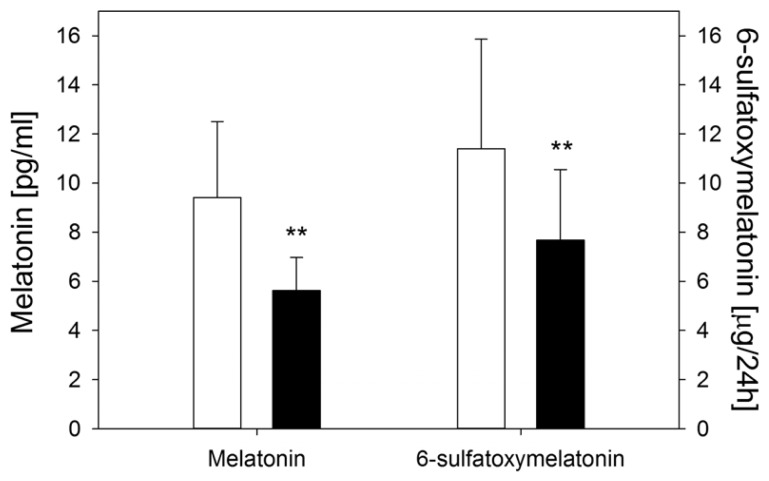
Fasting serum melatonin levels and urinary excretion of 6-hydroxymelatonin sulfate (6-HMS) (μg/24 h) in control subjects (white bars) and patients with continuous epigastric pain syndrome (black bars). *n* = 30 in either group; error bars denote standard deviation; **p < 0.01 compared with control.

The serum melatonin level on fasting (09:00 a.m.) was (9.41 ± 2.84) pg/mL in healthy subjects, whereas it was lower in CEPS patients: (5.62 ± 1.62) pg/mL (*p* < 0.01) (Figure 3). Urinary 6-HMS excretion in the control group was (11.40 ± 4.49) μg/24 h and in the CEPS group it was significantly lower: (7.68 ± 2.88) μg/24 h (*p* < 0.01) (Figure 3).

**Figure 4:**
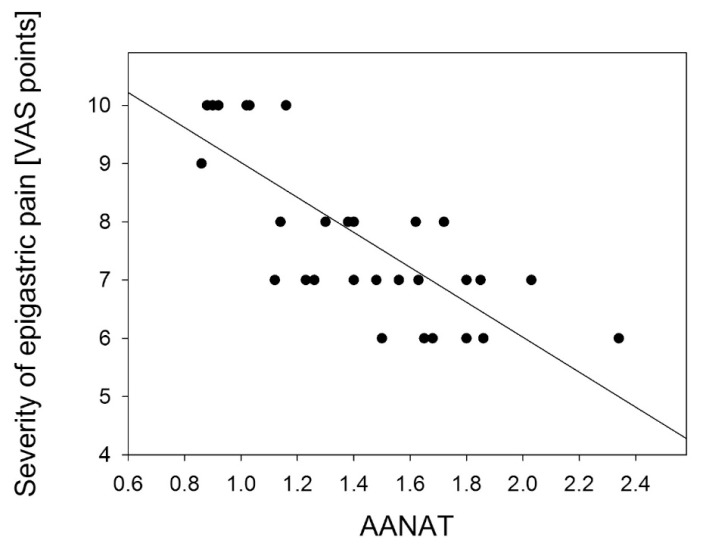
Correlation between arylalkylamine N-acetyltransferase (AANAT) expression assessed as relative quantity of mRNA of AANAT and hypoxanthine phosphoribosyltransferase (reference) by RT-PCR in antral gastric mucosa and the severity of epigastric pain assessed by Visual Analog Scale (VAS) in patients with continuous epigastric pain syndrome (*n* = 30). Regression line was calculated by means of the last square method, the *R* value was equal to −0.778, *p* < 0.001.

**Figure 5:**
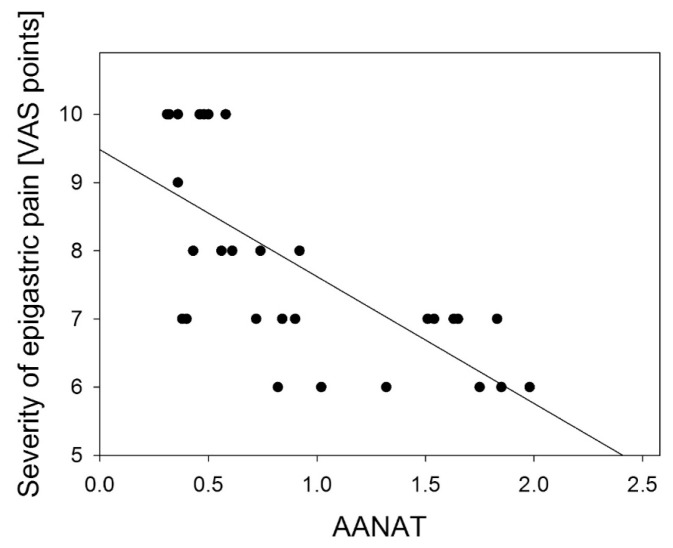
Correlation between arylalkylamine N-acetyltransferase (AANAT) expression assessed as relative quantity of mRNA of AANAT and hypoxanthine phosphoribosyltransferase (reference) by RT-PCR in body gastric mucosa and the severity of epigastric pain assessed by Visual Analog Scale (VAS) in patients with continuous epigastric pain syndrome (*n* = 30). Regression line was calculated by means of the last square method, the *R* value was equal to −0.697, *p* < 0.001.

**Figure 6:**
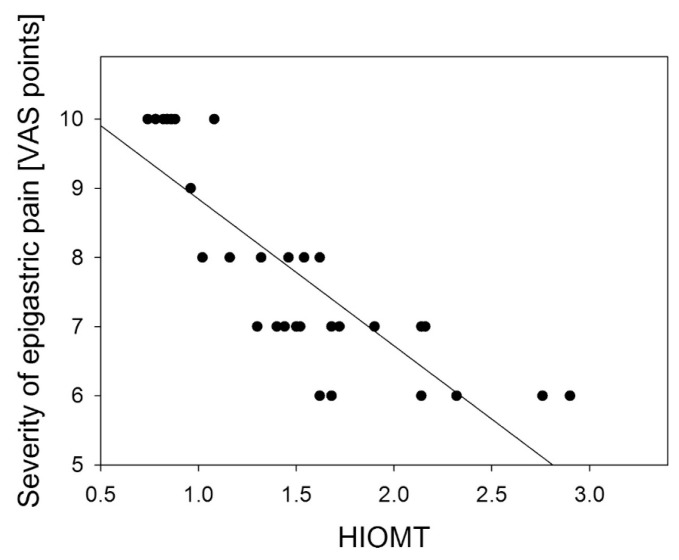
Correlation between hydroxyindole-*O*-methyltransferase (HIOMT) expression assessed as relative quantity of mRNA of HIOMT and hypoxanthine phosphoribosyltransferase (reference) by RT-PCR in antral gastric mucosa and the severity of epigastric pain assessed by Visual Analog Scale (VAS) in patients with continuous epigastric pain syndrome (*n* = 30). Regression line was calculated by means of the last square method, the *R* value was equal to −0.833, *p* < 0.001.

**Figure 7:**
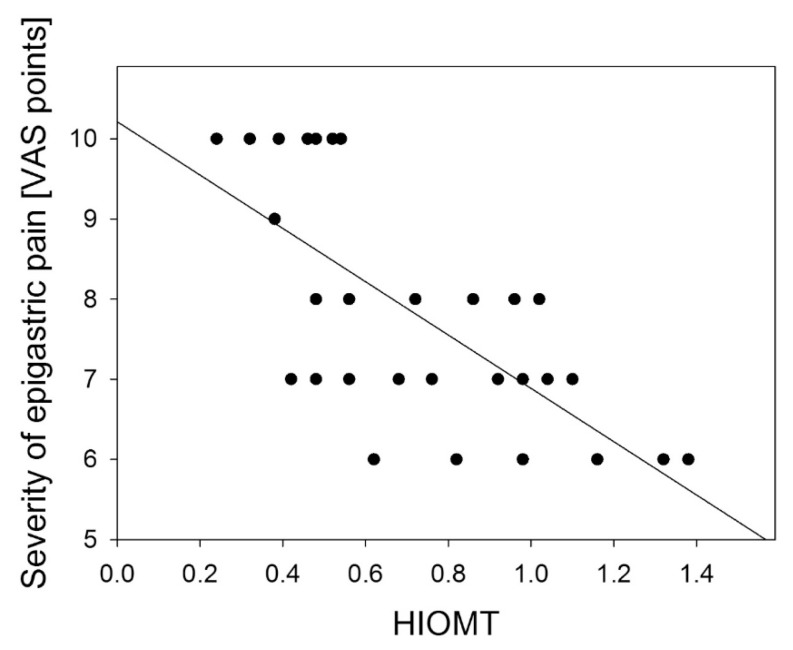
Correlation between hydroxyindole-*O*-methyltransferase (HIOMT) expression assessed as relative quantity of mRNA of HIOMT and hypoxanthine phosphoribosyltransferase (reference) by RT-PCR in body gastric mucosa and the severity of epigastric pain assessed by Visual Analog Scale (VAS) in patients with continuous epigastric pain syndrome (*n* = 30). Regression line was calculated by means of the last square method, the *R* value was equal to −0.698, *p* < 0.001.

A negative correlation was found between the severity of epigastric pain and AANAT and HIOMT expression level in the gastric antrum: *R* = −0.778 (*p* < 0.001) (Figure 4); *R* = −0.833 (*p* < 0.01) (Figure 5) and in the gastric body: *R* = −0.697 (*p* < 0.01) (Figure 6); *R* = −0.698 (*p* < 0.001) (Figure 7).

**Figure 8:**
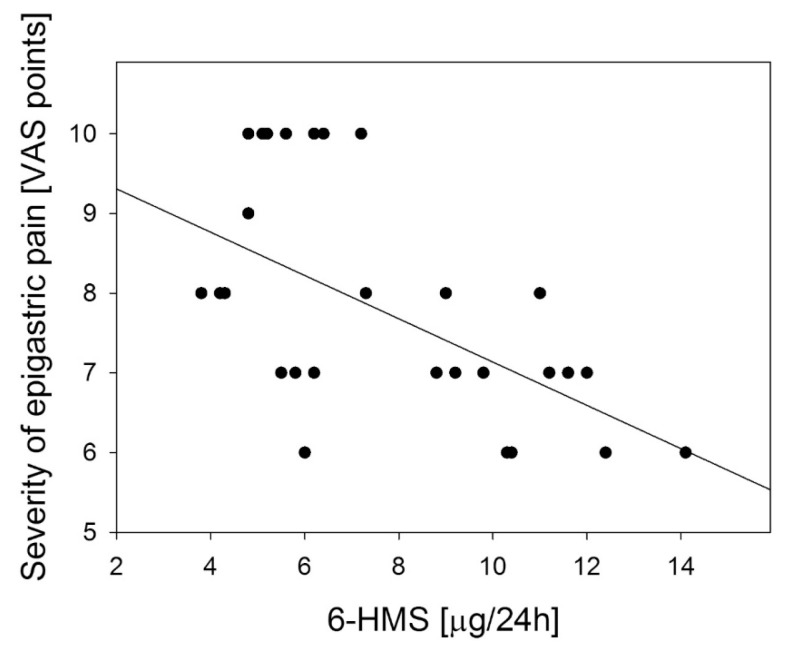
Correlation between urinary 6-hydroxymelatonin sulfate (6-HMS) in body gastric mucosa and the severity of epigastric pain assessed by Visual Analog Scale (VAS) in patients with continuous epigastric pain syndrome (*n* = 30). Regression line was calculated by means of the last square method, the *R* value was equal to −0.698, *p* < 0.001.

**Figure 9:**
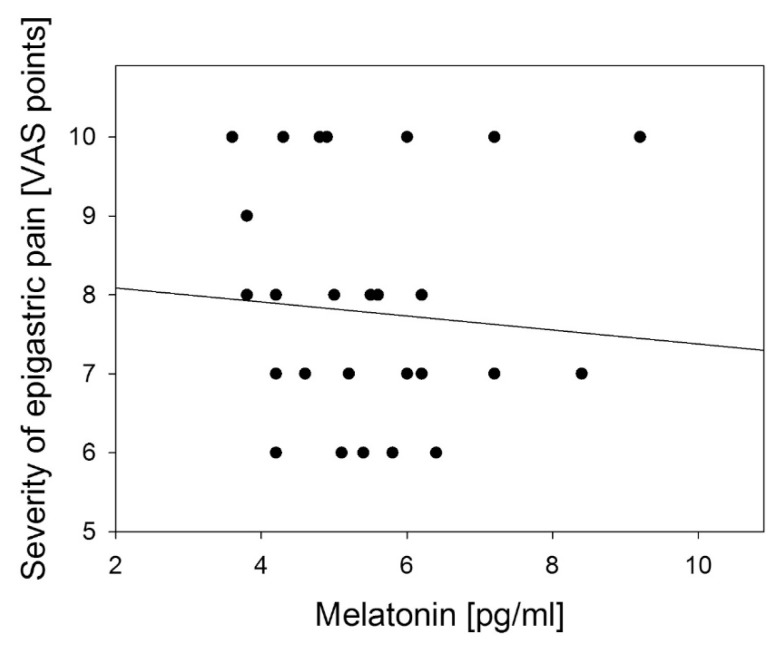
Correlation between fasting serum melatonin level and the severity of epigastric pain assessed by Visual Analog Scale (VAS) in patients with continuous epigastric pain syndrome (*n* = 30). Regression line was calculated by means of the last square method, the *R* value was equal to −0.698, *p* < 0.001.

Similar, but weaker correlation was observed between the severity of epigastric pain and urinary 6-HMS excretion: *R* = −0.536, (*p* < 0.01) (Figure 8) and no correlation with melatonin serum level: *R* = −0.082 (*p* > 0.05, Figure 9).

Melatonin has many beneficial properties. First of all, it displays antioxidant activity, protecting cells against reactive oxygen species [24,25]. We showed previously that MEL decreased oxidative DNA damage in gastrocytes [26]. It was suggested that melatonin inhibited HCl secretion, but stimulated the secretion of bicarbonates in the duodenum [27,28]. It may prevent the development of gastric ulcers by enhancing the protective properties of the mucosal barrier [16,29]. Melatonin manifests anti-inflammatory activity by inhibiting the production of proinflammatory cytokines and has immunomodulatory activity [30–33]. Moreover, it displays a relaxation action on smooth muscles in the GI tract [34–36]. The indole structure blocks the nicotinic acetylcholine receptors on the nerve endings of the submucosal plexi and activates afferent vagal fibres via increased release of cholecystokinin and activation of its receptors [37–39].

Beneficial properties of melatonin have been exploited in the treatment of GI tract diseases. It was demonstrated that MEL and l-tryptophan diet supplementation diminished the symptoms of esophageal reflux disease [40]. It was observed that the inclusion of MEL in therapy of gastroesophaegal reflux disease (GERD) resulted in stopping IPPs in maintaining remission of the disease [41]. Efficacy of melatonin in GERD therapy was confirmed both singly and in combination with omeprazole [42]. A beneficial effect of melatonin on gastric mucosa in patients with duodenal ulcer disease was noted [43]. We demonstrated the usefulness of MEL in the treatment of ulcer-like dyspepsia [44]. There have also been numerous reports about the beneficial effects of melatonin administration in patients with functional and inflammatory intestinal diseases [45–48].

Our study has several limitations with probably the most serious being the small population enrolled in our research. The next was that we estimated the intensity of abdominal pain with a rather rough scale, based on self-evaluation of average intensity experienced by the patients during the previous three months. Moreover, complex mechanisms underly gene expression and firm conclusions cannot be drawn on the basis of the mRNA level.

The results of our study suggest an involvement of melatonin in the pathogenesis of epigastric pain in patients with functional dyspepsia. In the group of patients with this syndrome, fasting blood melatonin levels did not differ from those of the control subjects, but AANAT and HIOMT expression in gastric mucosa were lower compared to the controls. Moreover, circadian urinary excretion of 6-HMS was lower than in healthy subjects. Low expression of this enzyme in gastric mucosa may affect several gastric secretory and motor functions as well as visceral sensation. Negative correlation between the level of AANAT and HIOMT expression and severity of pain may confirm this suggestion. However, melatonin may play an important role in visceral nocinception by exerting antinociceptive effects *via* a supra-spinal process associated with the central opioidergic system [49].

According to the Rome III criteria, the diagnosis of functional dyspepsia is based only on the analysis of dyspeptic symptoms reported by the patients. Thus, the objective causes of this disease are still unknown. The results obtained indicate that disturbed synthesis of melatonin in gastric mucosa and its decreased paracrine effect in this organ might play a role in the pathogenesis of epigastric pain.

## 3. Experimental Section

### 3.1. Patients

The study included 30 patients, 21 women and 9 men, aged 21–49 years (mean 32.2 ± 12.3 years) with functional EPS hospitalized in the Department of Gastroenterology, Medical University of Lodz, Lodz, Poland, due to reported GI complaints. The control group was comprised of 30 sex-matched individuals, aged 19–41 years (mean 29.8 ± 8.3 years). The control group was recruited from patients with excluded EPS, who met the inclusion criteria. No chronic gastritis cases were detected in either group as evaluated by gastroscopy and biopsy.

Symptoms of minimum 6-months duration and no improvement after spasmolytic, prokinetic and antacid drugs were the inclusion criteria. The severity of epigastric pain was estimated once with the Visual Analog Scale (VAS; 1–10 points) as the average from the previous three months. The mental state of the patients was also evaluated by psychiatrists to exclude psychiatric disorders.

### 3.2. Study Design and Procedures

Endoscopy of the upper part of the GI tract, gastric mucosa histopathology, abdominal ultrasound, laboratory tests: the blood cell count, CRP concentration, glucose, electrolytes, bilirubin, urea, creatinine, cholesterol, triglycerides, thyroid hormones and the activity of the following enzymes: aspartate aminotransferase (AST), alanine aminotransferase (ALT), gammaglutamyl-transpeptidase (GGTP), alkaline phosphatase (ALP), amylase and lipase were performed in all the patients enrolled in the study. *Helicobacter pylori* infection was excluded by histological examination and the urease breath test (UBT-13C).

Patients with organic, metabolic and psychic diseases as well as any pharmacological treatment and cigarette smokers were excluded from the study.

Seven days prior to the evaluations, all the medications were withdrawn and the same diet was introduced in all subjects with a similar daily amount of products rich in l-tryptophan. On the day of the study, the patients remained in the room with only red light from 9:00 p.m. until 07:00 a.m. and the same liquid diet was administered (Nutridrinks, Nutricia, Amsterdam, The Netherlands), 3 × 400 mL, 1800 kcal and 1500 mL of isotonic water. At the same time the 24-h urine collection was performed. Urine was kept at +4 °C. Immediately after the end of 24-h urine collection, the volume of urine was measured, centrifuged and the samples were frozen at −70 °C. The urinary 6-HMS concentration was measured by the ELISA method applying IBL antibodies (RE-54031, IBL, Hamburg, Germany) and Expert 99 MicroWin 2000 Reader (Biogenet, Jozefow, Poland). The results obtained were converted from nanograms per milliliter to micrograms/24 h.

Fasting serum melatonin concentration (picograms per milliliter) was determined by the ELISA method using IBL Kit (RE 59021). Blood was drawn from the basilic vein at 09:00 a.m. Material for histological and genetic examinations was collected from the antral part (4 biopsies) and upper part of gastric body (4 biopsies) directly after blood drawing. The level of mRNA was estimated with RT-PCR and 50 mg of gastric tissues were used for this purpose. Total RNA was isolated with trizol (Giboco, Darmstadt, Germany) reagents and then purified with DNase using Quiagen RNeasy Mini Kit. The quantity and quality of RNA were estimated spectrophotometrically. The obtained extract was used as a matrix in the analysis of gene expression. cDNA synthesis was performed with oligo(dT) 12–18 cycles in a thermocycler PTG-1000 MJ Research. cDNA obtained in reverse transcription was used for PCR reaction for fragments of the AANAT and HIOMT genes. The hypoxanthine phosphoribosyltransferase gene was a reference for the evaluation of the expression of both genes. The reaction products were separated on a 6%–10% polyacrylamide gel stained with ethidium bromide. Then, the products were subjected to densitometry to determine the reaction efficacy and the level of mRNA of genes of interest. The value of expression was compared to HPRT gene product to normalize the expression.

### 3.3. Ethics

The study was performed in accordance with the Declaration of Helsinki and with the principles of Good Clinical Practice. Written consent was obtained from each subject enrolled into the study and the study protocol was approved by the Bioethics Committee of the Medical University in Lodz (RNN/596/11/KB).

### 3.4. Statistical Analysis

The non-parametric Kruskal-Wallis test was used for the comparison of AANAT and HIOMT expression levels, melatonin concentration and urinary 6-HMS excretion. The Mann-Whitney test was applied for median comparison. The correlation between the above parameters and the severity of symptoms was estimated by the determination of the Pearson’s correlation coefficient and linear regression equation. Statistica and Excel software were used for statistical analysis.

## 4. Conclusions

Diminished production of melatonin in the gastrointestinal tract can play an important role in the pathogenesis of functional diseases of this organ, which justifies the usefulness of its application for therapeutic purposes.
